# Metabolic Characterization of All-Trans-Retinoic Acid (ATRA)–Induced Craniofacial Development of Murine Embryos Using *In Vivo* Proton Magnetic Resonance Spectroscopy

**DOI:** 10.1371/journal.pone.0096010

**Published:** 2014-05-09

**Authors:** Feifei Qin, Zhiwei Shen, Lihong Peng, Renhua Wu, Xiao Hu, Guishan Zhang, Shijie Tang

**Affiliations:** 1 Cleft Lip and Palate Treatment Center, Second Affiliated Hospital, Shantou University Medical College, Shantou, Guangdong Province, People's Republic of China; 2 Department of Medical Imaging, Second Affiliated Hospital, Shantou University Medical College, Shantou, Guangdong Province, People's Republic of China; 3 Department of Plastic and Burn Surgery, Guangzhou Red Cross Hospital, Guangzhou, Guangdong Province, People's Republic of China; National Research Council of Italy, Italy

## Abstract

**Aim:**

To characterize the abnormal metabolic profile of all-trans-retinoic acid (ATRA)–induced craniofacial development in mouse embryos using proton magnetic resonance spectroscopy (^1^H-MRS).

**Methods:**

Timed-pregnant mice were treated by oral gavage on the morning of embryonic gestation day 11 (E11) with all-trans-retinoic acid (ATRA). Dosing solutions were adjusted by maternal body weight to provide 30, 70, or 100 mg/kg RA. The control group was given an equivalent volume of the carrier alone. Using an Agilent 7.0 T MR system and a combination of surface coil coils, a 3 mm×3 mm×3 mm ^1^H-MRS voxel was selected along the embryonic craniofacial tissue. ^1^H-MRS was performed with a single-voxel method using PRESS sequence and analyzed using LCModel software. Hematoxylin and eosin was used to detect and confirm cleft palate.

**Result:**

^1^H-MRS revealed elevated choline levels in embryonic craniofacial tissue in the RA70 and RA100 groups compared to controls (P<0.05). Increased choline levels were also found in the RA70 and RA100 groups compared with the RA30 group (P<0.01). High intra-myocellular lipids at 1.30 ppm (IMCL13) in the RA100 group compared to the RA30 group were found (P<0.01). There were no significant changes in taurine, intra-myocellular lipids at 2.10 ppm (IMCL21), and extra-myocellular lipids at 2.30 ppm (EMCL23). Cleft palate formation was observed in all fetuses carried by mice administered 70 and 100 mg/kg RA.

**Conclusions:**

This novel study suggests that the elevated choline and lipid levels found by ^1^H-MRS may represent early biomarkers of craniofacial defects. Further studies will determine performance of this test and pathogenetic mechanisms of craniofacial malformation.

## Introduction

Craniofacial morphogenesis, an intricate developmental process, begins with the synchronized development of head primordia, which involves several organizing centers located in the neural ectoderm, axial mesendoderm, and the cranial neural crest [1]. The differentiation and spatial patterning of these tissues must occur before they can be successfully integrated [2]. Genetic disorders, environmental insults, or the combination of both can result in craniofacial malformations [3]. Craniofacial malformations are involved in three fourths of all congenital birth defects in humans [2]. Cleft lip and palate (CLP) is the most common birth defect in human craniofacial development, causes considerable morbidity to affected children, and imposes a substantial financial risk and a concomitant societal burden for families [4]. Defects arise early in embryological development, and have a complex etiology which has limited the identification of specific etiologic factors [5], [6]. Maternal conditions during pregnancy appear to play an important role in the formation of CLP, and vitamin deficiency and teratogens may increase risk for oral clefts [7]–[9].

All-trans retinoic acid (RA), an endogenous metabolite of vitamin A, is required for normal pattern formation during embryogenesis [10]. Conversely, abnormally high concentrations in both experimental animals and humans result in fetal malformations, including cleft palate [10], [11]. In murine embryos, all-trans RA has been shown to produce craniofacial malformations when treatment occurs between embryonic day 11 (E11) and E14.5. A single 100 mg/kg dose of all-trans RA to mice bearing E11 embryos has been shown to result in a 90–100% malformation frequency in mouse embryos [12].

Current strategies to study orofacial defects focus on related genotype and transcription factors, which only reveal part of what might be happening in a cell [13], [14]. Metabolism reflects the complex molecular interactions occurring in biological systems, and can be a good measure to understand the pathophysiology of craniofacial malformations [15].

Proton magnetic resonance spectroscopy (^1^H-MRS) is a versatile technique that can be used for measurement of metabolite levels and chemical reaction rates, and studies of bioenergetics without the need for invasive procedures such as biopsy [16]. ^1^H-MRS uses a static magnetic field to temporarily align the nuclear magnetization of protons within the body. Radiofrequency pulses are then applied which give the protons sufficient energy to alter this alignment and, as the protons return to their original state, the resulting radiofrequency signal is detected [17]. MRS has the potential to become a vital tool for aiding the understanding of changes due to pathology in specific regions of the body, as well as for clinical diagnosis and treatment monitoring [18], [19]. MRS has been used widely in clinical studies for diagnosis, prognosis, classification, or response to the therapy, resulting in many advances in the development of modeling techniques, chemometrics, and ways to identify new biomarkers [20], [21].

Researchers have demonstrated the relationship between the change in endogenous small molecular metabolites of maternal plasma and the incidence of cleft palate in fetuses [22]. However, there is no literature on the metabolite characteristics of craniofacial morphogenesis. In this study, we predicted that ^1^H-MRS method could identify the metabolic consequences of maternal environmental changes during pregnancy and illustrate the relationship between changes in maternal environment and development of craniofacial tissue. To test this prediction, we first assessed the feasibility of undertaking 1H MRS of craniofacial tissue in vivo, and then used ^1^H-MRS to characterize metabolites in fetal craniofacial tissue within pregnant mice induced by RA to trigger cleft palate formation in the embryos. The analyses demonstrated the relationship between the changes in endogenous small molecular metabolites of the craniofacial structure and the incidence of cleft palate in embryos.

## Materials and Methods

### Ethics

This study was approved ethically by the Animal Ethics Committee of Shantou University Medical College.

### Animal Husbandry

Kunming (KM) mice purchased from the Medical Animal Center of Medical College of Shantou University (Guangzhou, China) were housed in the Transformation Center in the Second Affiliated Hospital of Shantou University. They were maintained under a 12/12-house light/dark cycle, with standard food and tap water ad libitum. The female mice were mated with males with similar weights and ages. One virgin female mouse was assigned randomly to a single male mouse. Presence of a vaginal plug was assessed after overnight mating, and gestational stage was estimated by defining the morning of that day as embryonic gestation day (E) 0.5.

### Drug administration

Pregnant mice (n = 19) were randomly divided into four groups (three experimental groups and one control group). To evaluate the in vivo effect of exogenous RA, a single oral gavage of 30, 0 and 100 mg/kg RA suspended in 200 µl of sesame oil was administered to pregnant females at E11. All work with RA was conducted under dim yellow light to prevent photo-oxidation. Controls were treated with the equivalent amount of the carrier according to their weight.

### *In vivo* MRI studies

MRI experiments were performed with a 7.0 T animal MR imaging system (Agilent, 7T/160/AS, U.S.A) equipped with a 95/63 mm quadrature birdcage coil for transmitting and receiving radio frequence signal. Mice were maintain anesthetized with isoflurane (3% for induction, 1–2% for maintenance, Abbott Labratories Ltd, U.K.), which was mixed with oxygen (1 liter/min) and delivered through a nasal mask, allowing for spontaneous respiration. Once anesthetized, the animals were placed in a supine position on the center of the horizontal bore magnet for MRI/MRS scanning. Body temperature was maintained at approximately 35–37°C using a plastic membrane.

Three planes location imaging was first acquired with a gradient echo sequence for abdominal position. Subsequently, a fast spin echo multi-slice (fsmse) T2-weighted imaging in axial and sagittal position was acquired with the following parameters: field of view  = 23 mm×19 mm, matrix size  = 256×256, repetition time (TR)  = 2000 ms, effective echo time (TE)  = 31.58 ms, slice thickness  = 2 mm, slice gap  = 0.5 mm, and acquisition time  = 4 min 20 seconds.

### ^1^H-MRS

A PRESS pulse sequence (TE/TR 144/1500 ms, 96 averages) with VAPOR water suppression was used for ^1^H-MRS acquisition. A 3×3×3 mm voxel of interesting (VOI) was located on the craniofacial region of the embryo. Voxel selection was confirmed to be limited to within the craniofacial region, using the range of orthogonal fast spin echo slices. Outer volume suppression was used around the voxel to further prevent any non-craniofacial contaminant signals. A manual shim over the second-order ge3dshim automatic shim was applied over the selected voxel to counter any local inhomogeneities in the magnetic field. In our experience, the craniofacial unit does not significantly move out with our selected voxel dimensions during either maternal breathing, or fetal motion. The resulting raw spectral data were exported to an external workstation and MRS analysis was used to assess the craniofacial metabolism.

### Data analysis

Lipid and choline were quantified using the LCModel software package (Version 6.3-1B, LCMODEL Inc. CA). The quantification algorithm of the LCModel applies linear combinations to calculate the best fit of the experimental spectra to the model spectra. In this study, a special type of ‘muscle-5’ was used for muscle spectra analysis. The metabolite resonance area to the unsuppressed water resonance area was calculated in the same voxel by LCModel. The tissue water concentration served as the internal standard, was estimated as 42,223 mmol/L. Absolute concentrations of choline, intra-myocellularlipid (IMCL) were acquired with T1 and T2 relaxation effects correction.

The spectra were inspected for residuals and large artefacts in the spectral region of interest. In order to avoid any bias towards high concentrations, the data were discarded if the reported signal-to-noise ratio (SNR) of spectra from LCModel was less than 5, or if the standard deviation (SD) of a metabolite was more than 20%. The data were also discarded if shown as missing values by boxplot.

### Histological sections

Pregnant females (n = 19) were sacrificed by cervical dislocation and embryos (n = 211) were harvested at E17.5. At the same time, a stereomicroscope was used to determine the number of fetuses and gross malformations. After removal of the mandibles, embryonic palates were calculated using stereomicroscope (10×) and divided by morphology in four groups. Target palates were processed into paraffin wax. Histological sections of embryonic palates were cut and stained with haematoxylin and eosin (HE).

### Statistical analysis

All data were presented as a mean ± standard deviation (SD). Statistical significance was calculated using Mann-Whitney Test and was accepted at P≤0.05. All analyses were performed using SPSS software (version 16.0, IBM SPSS Statistics, Inc, United States).

## Results

### Magnetic Resonance Imaging (MRI)

**Figure 1:**
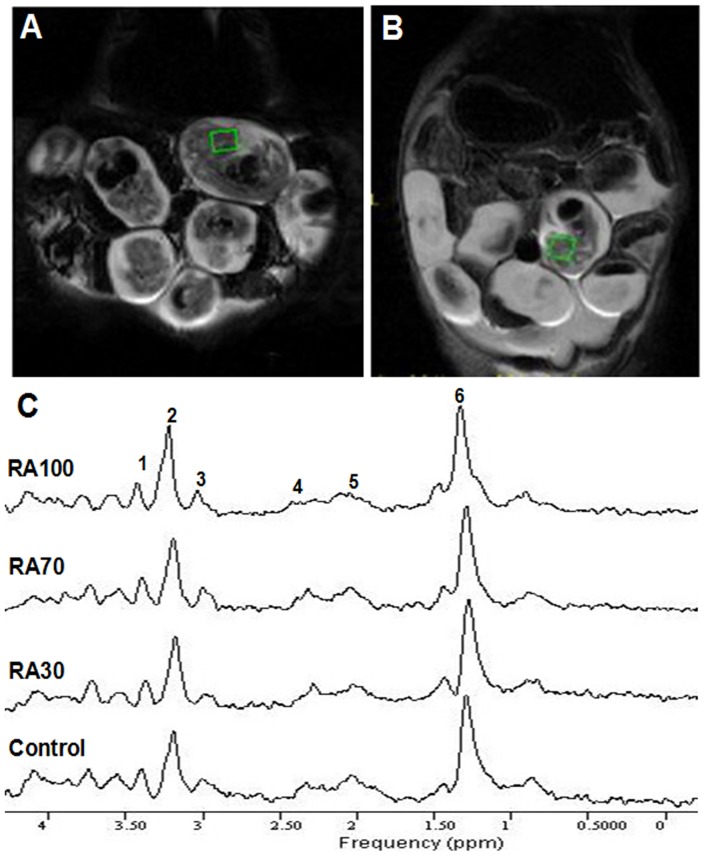
MRI and ^1^H-MRS of mouse fetuses. A. Magnetic resonance imaging (MRI) of E14.5 mouse embryos at a dose of 70 mg/kg RA. Volumes of interest (VOI)  = 3.0×3.0×3.0 mm3. B. MRI of E14.5 mouse embryos at a dose of 100 mg/kg RA. Hyperintense regions were observed in the imaging. There were parts of or no embryos in the hyperintense regions. VOI  = 3.0×3.0×3.0 mm^3^. C. In vivo proton magnetic resonance spectroscopy (^1^H-MRS) obtained from the control and three experimental groups after RA treatment. Resonances were readily identified by the LCModel software. 1. Taurine; 2. Choline; 3. Creatine; 4. Extramyocellular lipids (EMCL23); 5. Intramyocellular lipids (IMCL21); 6. IMCL13.

Using a fast spin-echo MRI sequence, high resolution MR images of E14.5 mouse embryos could be acquired. Figure 1A represents an in vivo coronal T2-weighted MR imaging of a pregnant mouse harboring E14.5 embryos, following treatment with RA at 70 mg/kg. Hyperintense regions were observed in the imaging of the mouse treated with 100 mg/kg RA (Figure1B). We determined that some fetuses were reabsorbed due to toxicity.

### Proton Magnetic Resonance Spectroscopy (^1^H-MRS)

In vivo localized 1H magnetic resonance spectra were obtained from the control and three experimental groups after RA treatment. A 3 mm×3 mm×3 mm volume of interest (VOI) was used in every fetus. A typical set of in vivo 1H-MR spectra acquired from the craniofacial tissues in each group is shown in Figure 1C. Resonances were readily identified by the LCModel software. The regions of the significant metabolite signals usually ranged from δ 0.8–3.8 ppm, including lactate (δ 1.32 ppm), IMCL13 (intra-myocellular lipids) (δ 1.30 ppm), IMCL21 (δ 2.10 ppm), EMCL23 (extra-myocellular lipids) (δ 2.30 ppm), total choline (δ 3.19 ppm), total creatine (δ 3.04 ppm), and taurine (δ 3.43 ppm).

**Figure 2:**
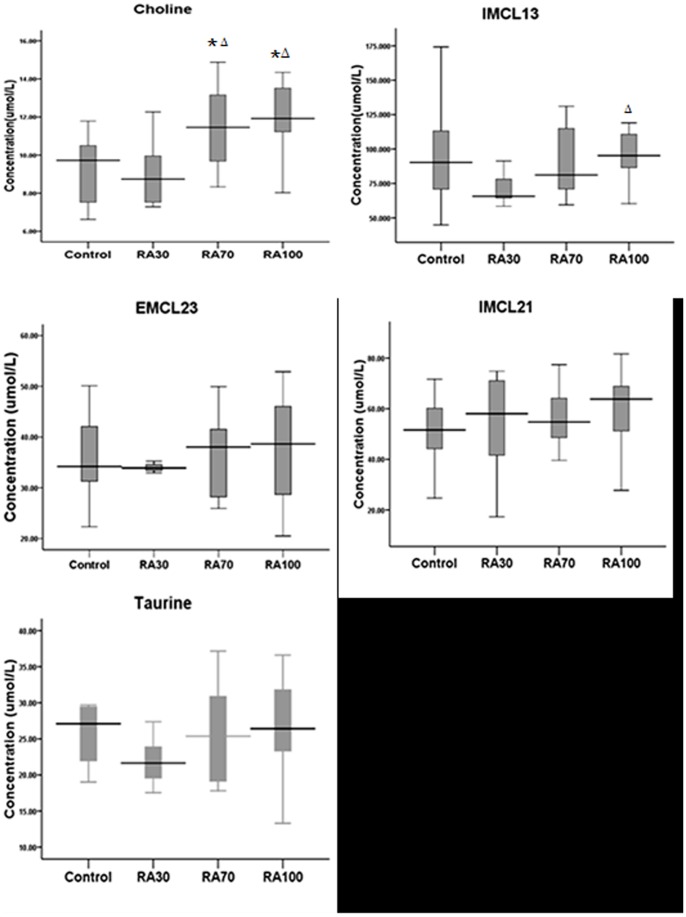
The boxplot from ^1^H-MRS of embryonic craniofacial tissues' metabolite concentrations. The boxplot from ^1^H-MRS shows quantification of IMCL13, choline, taurine, IMCL21, EMCL23 concentrations in embryonic craniofacial tissues of pregnant mice. Values are mean ± standard deviation (SD). Metabolite concentrations were compared respectively. *Significant difference compared to control (P<0.05); △Significant difference compared to RA30 (P<0.05).

1H-MR spectroscopy revealed that compared with the control group, elevated choline levels (umol/L) were observed in embryonic craniofacial tissues in the RA70 group (11.51±1.89 for RA70, n = 14 vs. 9.30±1.66 for control, n = 10, Z = −2.342, P = 0.019) and RA100 group (12.04±1.91 for RA100, n = 11 vs. 9.30±1.66 for control, n = 10, Z = −2.958 P = 0.002) by 1H-MR spectroscopy. An increase in choline levels was also found in both RA70 and RA100 groups compared with the RA30 group (12.04±1.91 for RA100, n = 11 vs. 9.27±1.85 for RA30, n = 9, Z = −2.621, P = 0.007; 11.51±1.89 for RA70, n = 14 vs. 9.27±1.85 for RA30, n = 9, Z = −2.268, P = 0.023). High levels of IMCL13 (umol/L) in RA100-treated mice compared to RA30 were found (71.37±11.14 for RA30, n = 9 vs. 95.84±16.22 for RA100, n = 14, Z = −3.024, P = 0.002). There were no significant changes in taurine, IMCL21, and EMCL23 (Table 1). Figure 2 shows a boxplot of the metabolites in every group.

**Table 1:** Metabolite concentrations determined by in vivo 1H-MR spectroscopy of embryonic craniofacial tissues.

Metabolite	Control	RA30	RA70	RA100
IMCL13	93.90±37.55	71.37±11.14	89.86±26.03	95.84±16.22△
Choline	9.30±1.66	9.27±1.85	11.51±1.89* △	12.04±1.91* △
Taurine	25.71±4.20	21.95±3.41	25.34±6.48	26.47±6.82
IMCL21	51.04±14.47	53.48±21.53	56.48±10.59	59.64±15.96
EMCL23	36.19±9.26	34.02±1.16	36.34±7.81	37.29±10.59

The concentration unit of metabolites is µmol per litre (umol/L).

Abbreviations: IMCL, intramyocellular lipids; EMCL, extramyocellular lipids.

All fetuses measured at E14.5, values are mean ± standard deviation (SD).

*Significant difference compared to control (P<0.05).

△ Significant difference compared to RA30 (P<0.05).

### Incidence of Cleft Palate in Four Groups

Nineteen KM pregnant mice were examined, and 211 embryos were collected, including 156 embryos from RA-treated group, and 55 embryos from the control group (Table 2). Each pregnant mouse had multiple embryos. There was at least one CP embryo in each pregnant mouse in the RA treatment group, while there were no CP embryos in the control group. The incidence of CP in the RA70 and RA100 treatment groups was 100%, and the incidence of CP in the RA30 group was 40%.

**Table 2:** Comparison of the incidence of cleft palate in the embryos between four groups.

	Dam n	Total Fetus n	Death n	Cleft Palate n	Ratio (%)
Control	5	55	0	0	0
RA30	4	40	0	16	40
RA70	5	61	2	59	100
RA100	5	55	20	35	100

**Figure 3:**
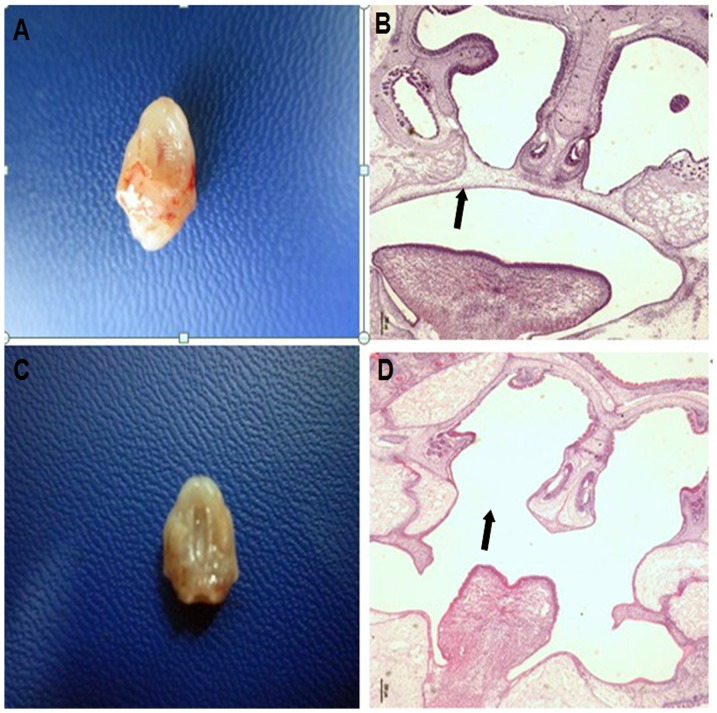
Fusion or lack of fusion of embryonic palatal shelves. A: Fused embryonic palatal shelves. B: A failure to fuse embryonic palatal shelves. C: Fused embryonic palatal shelf in HE stain. D: A failure to fuse of embryonic palatal shelves in HE stain.

The morphology and histology were examined (Figure 3). “A” and “C” show fusion of the palate shelves, while “B” and “D” show a failure of the palate shelves to fuse.

## Discussion

The aim of this study was to identify early changes in metabolite concentrations associated with RA-induced cleft palate with a view to provide biomarkers for craniofacial defects. To this end, in vivo ^1^H-MRS was used in the study as a noninvasive method of investigating changes in response to RA. We determined that noninvasive quantitative measurements of choline and lipid by ^1^H-MRS were important for the assessment of facial structures. In the current study, total Cho levels in the fetal craniofacial structures were found to increase following administration of 70 and 100 mg/kg RA. We interpret the observed increase in the levels of choline in RA-induced mice to be an effect of the disease process. The data in this study show the feasibility of measuring choline concentration in craniofacial tissue in vivo using 7.0 T MR acquisition and quantification methods.

RA, as an important regulator of embryogenesis, regulates proliferation, differentiation, and apoptosis during the morphogenesis of embryonic structure. [11], [12], [23] This is a putative biological mechanisms underlying RA-induced CLP. Overexposure to RA can cause congenital malformations such as CLP. Depending on the timing of exposure, excess RA might disturb all stages of palatogenesis. During shelf outgrowth, it may decrease mesenchymal proliferation and thus prevent tissue expansion. In the next stage, RA may prevent shelf elevation by affecting the ECM composition and hydration, and during the actual fusion of the shelves, it may affect epithelial differentiation and apoptosis, which precludes the formation of a continuous palate. Growth of the palate shelves depends on the survival and continued proliferation of mesenchymal cells that originate from neural crest and mesodermal cells of the first pharyngeal arch [10], [24]. In the normal mouse embryo, palate shelves grow and elevate into a horizontal position and appose in the midline by E14.5. By E17.5, the process of fusion has completely finished. It was convenient to use morphology for embryo analysis. So we chose to collect samples on E17.5 [25].

The choline peak resonating at 3.2 ppm, seen in the in vivo ^1^H-MRS, is from total choline and consists of phosphocholine, glycerophosphocholine, and free choline [26]–[29]. Choline phospholipid metabolism comprises a complex network of biosynthetic and breakdown pathways, with one or more enzymes acting per pathway. Collectively, the MRS data presented in this study, combined with histologic analysis of craniofacial structures, supports the mechanism of action of RA. One interpretation is that RA toxicity could cause an increase in membrane precursors (phosphocholine and phosphoethanolamine) and subsequently lead to the observed increase in membrane degradation products (glycerophosphoethanolamine and glycerophosphocholine). Phosphocholine is a precursor as well as a metabolic breakdown product of the major membrane component phosphatidylcholine [30]. A high degree of membrane degradation will lead to high choline levels. The molecular and genetic alterations in craniofacial development,as well as changes in signaling pathways, act on the expression and post-translationally regulate activity levels of these enzymes, thereby generating changes in the overall choline metabolite profile. Choline-containing metabolites are associated with cell membrane phospholipid metabolism. Therefore, changes in their concentration detected using in vivo ^1^H-MRS of craniofacial tissues treated with RA are indicative of changes in cell membrane metabolism. The increase in total choline following RA treatment suggests RA causes an increase in cell membrane turnover.

In the last 10 years, the choline metabolite profile has been increasingly used as an adjunct for diagnosis of primary malignant tumors of the brain, prostate, and breast [31]. The choline peak is also thought to reflect cellular density. For instance, an increase in the choline peak is associated with an increase in membrane breakdown or turnover, myelination or inflammation and has been observed in demyelinating diseases and in tumours. Thus, the elevation is increasingly being used as an endogenous biomarker of cancers. The enzymes and pathways resulting in these distinct alterations in phosphocholine and total choline may provide novel molecular targets for specific, targeted anticancer therapies. ^1^H-MRS can give spectra comparable with those obtained in the present study when used on appropriately sited tumors in patients. For example, in vivo ^1^H-MRS has recently been successfully used in the clinic to determine total choline concentration of locally advanced breast cancer before and after neoadjuvant chemotherapy to predict response [32].

We have identified a previously unknown association between choline metabolism and RA-induced craniofacial abnormalities. To resolve the components of the total choline signal and further characterize the differences detected in vivo, we will perform high-resolution 1H-MR spectroscopy of tissue extracts in the future. Moreover, we will further study the metabolic patterns that may represent new noninvasive biomarkers and targets in the abnormal development of craniofacial structure, choline kinase mRNA and protein levels, for example.

In our present study, an increased embryonic lipid content (at 1.30 ppm) following dosing at 100 mg/kg, compared to 30 mg/kg, was observed. ^1^H-MRS has been successfully used to quantify lipid content, which is commonly divided into two compartments, IMCL and EMCL. While EMCL is relatively metabolically inert, IMCL can be utilized by mitochondria, making it the preferred form of lipid storage. In our study, the amount of IMCL13 significantly changed in the high dose group. Excess RA disturbs palatogenesis, which affects mesenchymal and epithelial differentiation, and apoptosis. Coincident with the onset of differentiation and apoptosis are altered lipid packing of the lipid bilayer, membrane blebbing, and decreased membrane microviscosity, which all could change the plasma membrane fluidity [33]. Another mechanism could involve an increase in the amount of fatty acids with the lipid bilayer. Thus, the complex process results in large lipid resonances (at 1.30 ppm) by ^1^H-MRS in vivo.

This study confirmed the feasibility of RA-induced animal model again. Normally, the incidence of cleft palate in KM natural mice is zero. We injected sesame oil in control samples in order to keep the same stimulating circumstances as in the RA-induced group. Regarding cell membrane turnover and cell proliferation and apoptosis, a significant effect is detected after administration of a 70 mg/kg dose of RA. A 100 mg/kg dose also shows the same change, but proved to be too toxic for the mice by MRI and anatomy, and the 30 mg/kg dose did not cause malformation in all fetuses. Hence, the rate of CLP as assessed by this work appears consistent with the results of all earlier study [12], that doses of 70 mg/kg or higher induce craniofacial malformation.

To our knowledge, this is the first report of in vivo 1H-MR spectroscopy for craniofacial development and the first report in which quantitative choline and lipid measurements are made by ^1^H-MRS. However, there is considerable individual variation in the associations. In particular, the assessment of individual treatment responses in vivo may benefit from combining with ^1^H-MRS. These promising findings were obtained in a small group of mice, and thus, a large study may also provide information about the possibility of using spectroscopy to predict craniofacial malformation and determine therapeutic responses. Moreover, MRS can also lead to new hypotheses regarding pathogenetic mechanisms and to new insights and a challenging new area in the study of craniofacial malformation in the future.
